# Don’t drink and drive, it’s a prime: Cognitive effects of priming alcohol-congruent and incongruent goals among heavy versus light drinkers

**DOI:** 10.1177/1359105320934166

**Published:** 2020-06-12

**Authors:** Zachary W Petzel, Jeffrey G Noel

**Affiliations:** 1 Queen’s University Belfast, UK; 2 Univeristy of Missouri – St. Louis, USA

**Keywords:** alcohol, alcohol expectancies, college students, drinking behaviour, goal priming, implicit attitudes

## Abstract

The present experiment assessed implicit alcohol motivations and explicit alcohol expectancies following the interaction between alcohol-congruent (i.e. social drinking) versus incongruent (i.e. driving safety) goal primes and recent drinking habits among college students (*n* = 176). Heavy drinkers exhibited greater implicit alcohol approach and explicit tension reduction expectancies following social goal primes, while displaying greater implicit alcohol avoidance and explicit cognitive and behavioural impairment expectancies after driving safety goal primes. These findings indicate recent drinking habits interact with goal salience to influence explicit and implicit responses to alcohol, which has implications for the development of interventions to reduce college drinking.

Half of college students in the United States engage in heavy episodic drinking (HED), drinking four or more drinks for females or five or more drinks for males in one sitting (White and Hingson, 2013). Many researchers seek to develop interventions that reduce students’ HED by influencing cognitive and motivational precursors to high-risk alcohol use (Houben et al., 2010; Noel et al., 2019). A long-standing intervention target has been self-reported alcohol expectancies, anticipatory assessments of positive and negative outcomes related to alcohol use. Expectancies predict drinking behaviour among college students (Hasking et al., 2011), however, interventions which challenge explicit alcohol expectancies (e.g. demonstrating rewarding effects of drinking may also be experienced after a placebo; Lau-Barraco and Dunn, 2008) may not be effective in reducing long-term alcohol consumption (Scott-Sheldon et al. 2012). Dual-process models of cognition have given rise to alternative approaches for measuring anticipated positive and negative drinking outcomes, such as through behavioural tasks like the Implicit Association Test (IAT) which predict unique variance in drinking behaviour (Reich et al., 2010). Interventions and manipulations targeting automatic or implicit cognitions, like those assessed in the IAT, may enhance interventions to promote health behaviours (Cristea et al., 2016; Papies, 2016).

Health behaviours linked to habit formation (e.g. alcohol use) may be particularly resistant to change via manipulations targeting only self-reported or explicit cognitions such as expectancies (Papies, 2016). An understudied manipulation which targets both explicit and implicit cognitions and may be useful in reducing HED among college students is goal priming. After a goal is activated, cognitions shift to align with goal-related behaviours to facilitate change (Ferguson and Porter, 2010). For example, completing a sentence unscrambling task or questionnaire referencing socialising goals promotes greater social-related cognitions (Ferguson, 2008), suggesting exposure to goal-related primes may impact subsequent behaviour. Whereas goal priming facilitates attitude change toward non-alcohol targets (Ferguson and Porter, 2010), little research has examined how priming alcohol-related goals may influence attitudes toward alcohol (Sheeran et al., 2005; Webb et al., 2012).

While a variety of manipulations are established to influence attitudes towards health behaviours (e.g. alcohol use; Lau-Barraco and Dunn, 2008; Noel et al., 2019), certain factors may reduce or promote their effectiveness. Regarding alcohol, previously learned associations and situational contexts may contribute to the effectiveness of college drinking interventions (Ham et al., 2013; Noel et al., 2019). For example, while many college students learn to associate drinking with negative outcomes, heavy drinkers discount these consequences relative to the rewards associated with alcohol (Wiers et al., 2005). Therefore, heavy drinkers exhibit stronger baseline alcohol-approach tendencies and associations compared to light drinkers (Field et al., 2008), suggesting interventions aiming to reduce college drinking may lead to more measurable change when targeted towards those engaging in HED. Further, college students exhibit more *negative* alcohol cognitions and associations in negative compared to positive (e.g. social) contexts (Ham et al., 2013), indicating a path toward reducing pro-alcohol cognitive bias. That is, college drinking interventions may benefit from making negative alcohol-related associations and contexts salient among heavy drinkers, who would typically discount these negative associations.

Goal priming may be useful in activating these negative associations to reduce HED among heavy drinkers. Activation of alcohol-congruent goals (e.g. socialising) promotes positive alcohol-related cognitions (Sheeran et al., 2005; Webb et al., 2012). However, relevant to potential drinking and health interventions, whether an alcohol-incongruent goal linked to negative alcohol-related outcomes and contexts (e.g. driving under the influence) activates negative alcohol cognitions is unclear. Thus, we aimed to inform potential drinking interventions through a preliminary investigation of how activating alcohol-congruent (e.g. socialising) versus alcohol-incongruent (e.g. driving safety) goals influence implicit alcohol motivations and explicit alcohol expectancies between heavy versus light college drinkers. We expected priming social drinking goals would promote positive alcohol cognitions, whereas priming driving safety goals would elicit negative alcohol cognitions. However, we expected goal activation would be most effective among heavy drinkers.

## Method

### Participants

Undergraduate students (*n* = 176; 71.5% female; *M*_age_ = 23.81, *SD*_age_ = 7.59) at a Midwestern University were recruited to participate in an experimental research paradigm, receiving course credit for their participation. All participants reported drinking alcohol within the past 30 days and identified as White/Caucasian (56%), Black/African American (28.6%), Latino/a (6.3%), or as another race (9.1%). Only 10.8% (*n* = 19) reported belonging to a fraternity or sorority. A sensitivity power analysis indicated 80% power to detect an approximately small to medium effect size (η^2^ = 0.04).

### Measures and procedure

After providing informed consent, participants self-reported demographics, as well as lifetime and recent drinking behaviour. Next, they were randomly assigned to goal priming conditions (social drinking, driving safety). After completing the priming task, participants completed measures of implicit alcohol motivation and explicit alcohol expectancies, with order of implicit and explicit measures counterbalanced between subjects.

#### Alcohol use and experiences questionnaire

Alcohol use was assessed using items adapted from the National Institute on Alcohol Abuse and Alcoholism (NIAAA) Task Force on Recommended Alcohol Questions (Bartholow et al., 2018; NIAAA, 2003). Participants were provided standard drink definitions (e.g. 12-ounce can) and asked 12 questions regarding alcohol use within various time intervals (e.g. 30 days, 3 months, lifetime) including reporting average number of drinking occasions (closed response; e.g. once a month, 2 to 3 times a month), average number of drinks consumed per occasion (free response), and maximum numbers of drinks recently consumed (free response).

#### Goal priming task

Participants completed a questionnaire which focused on socialising or driving safety (see Supplemental materials). In the social priming condition participants were asked to answer 14 questions rating the frequency with which they engaged in various activities with friends and/or family (e.g. going to concerts, going to happy hour), as well as two open-ended questions about the importance of friends and how common it is for adults to drink while socialising. Those assigned to driving safety answered 14 questions about how frequently they engage in safe driving behaviours (e.g. limiting distractions, using turn signals), as well as two open-ended questions about the importance of safe driving habits and how common it is for adults to drink alcohol and then drive an automobile. Social priming questions were adapted from Ferguson (2008), while additional items linked to alcohol and driving safety were created using similar methodologies to previous goal priming work (e.g. Sheeran et al., 2005).

#### Implicit alcohol motivation

The alcohol motivation IAT was modelled after Palfai and Ostafin (2003). Participants sorted pictures (i.e. soft drinks or alcoholic beverages) and words (i.e. approach-related or avoidance-related). In one set of critical trials, participants were instructed to sort alcohol images with avoidance-related words and soft drinks with approach-related words. In another set of critical trials, these pairings were switched. Order of critical trial blocks was counterbalanced. Reaction time-based D scores were calculated with positive scores indicating stronger alcohol-avoidance associations and negative scores suggesting stronger alcohol-approach associations (see Greenwald et al., 2003).

#### Explicit alcohol expectations

The comprehensive effects of alcohol (CEOA; Fromme et al., 1993) assesses anticipation of positive and negative drinking outcomes and consists of 38 items that make up four positive subscales (sociability, tension reduction, liquid courage, sexuality) and three negative subscales (cognitive/behavioural, risk/aggression, self-perception). Participants reported the likelihood they would experience the outcome described when drinking alcohol from 1 (*Disagree*) to 4 (*Agree*). Participants also reported the valence (positive or negative) of that outcome from 1 (*Bad*) to 5 (*Good*).

#### Ethical Approval

All measures and procedures were approved by the University’s institutional review board.

#### Data Analysis

Participants were classified as engaging in HED if they indicated consuming 5 or more (for males) or 4 or more (for females) alcoholic drinks on at least one occasion within the past 30 days (Hingson et al., 2009). Using these criteria, 48.3% of the recruited sample were classified as engaging in recent HED. Implicit alcohol motivation was analyzed using a 2 (goal activation: social, safety) × 2 (drinker status: non-HED, HED) factorial analysis of variance (ANOVA), with subsequent univariate ANOVAs used to probe significant interactions. Explicit alcohol expectancies were analyzed using a 2 (goal activation) × 2 (drinker status) multivariate ANOVA, performed separately on valence and expectancy estimates of the seven subscales of the CEOA. Subsequent factorial ANOVAs were used on individual estimates, with univariate ANOVAs used to probe significant interactions.

## Results

### Implicit alcohol motivation

**Figure 1.**
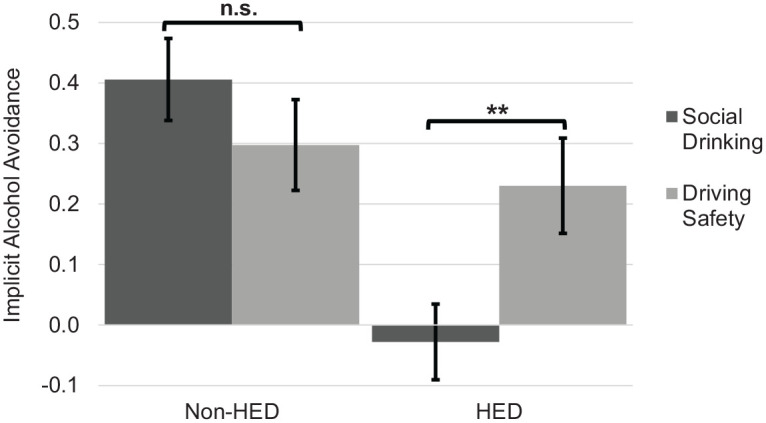
Implicit alcohol avoidance motivation as a function of goal activation (social, safety) and drinker status (non-HED, HED). Error bars represent standard error. **p < 0.01, *n.s.* = non-significant.

No significant main effect of goal activation on alcohol motivation emerged, *F*(1172) = 1.04, *p* = 0.309, η_p_^2^ = 0.006. However, a main effect of drinker status emerged, *F*(1172) = 11.65, *p* = 0.001, η_p_^2^ = 0.063, indicating HEDs demonstrated less alcohol avoidance (*M* = 0.09, *SD* = 0.50) compared to non-HEDs (*M* = 0.23, *SD* = 0.51). The interaction between goal activation and drinker status was significant, *F*(1172) = 6.23, *p* = 0.014, η_p_^2^ = 0.035. Among non-HEDs, there were no differences in alcohol motivations between social (*n* = 47) versus safety (*n* = 44) conditions, *F*(1172) = 1.13, *p* = 0.290, η_p_^2^ = 0.007. However, HEDs primed with social goals exhibited greater implicit alcohol approach (*n* = 45; *M* = −0.03, *SD* = 0.48) compared to safety goals (*n* = 40; *M* = 0.23, *SD* = 0.49), suggesting safety primes promoted alcohol avoidance in heavier drinkers, *F*(1172) = 5.97, *p* = 0.016, η_p_^2^ = 0.034 (see Figure 1).

### Explicit alcohol expectancies

No main effects emerged for valence or expectancy estimates, all *p*s > 0.220. The interaction between factors significantly affected expectancy, Wilks’ λ = 0.907, *F*(7166) = 2.42, *p* = 0.022, η_p_^2^ = 0.093, but not valence estimates, *p* = 0.089. Significant interactions emerged between goal activation and drinker status for cognitive/behavioural impairment, *F*(1172) = 5.92, *p* = 0.016, η_p_^2^ = 0.036 and tension reduction, *F*(1172) = 7.51, *p* = 0.007, η_p_^2^ = 0.042. HEDs in the driving safety condition reported significantly higher impairment expectations (*M* = 3.08, *SD* = 0.36) compared to the social condition (*M* = 2.81, *SD* = 0.60), *F*(1172) = 4.80, *p* = 0.030, η_p_^2^ = 0.027. Conversely, HEDs reported greater tension reduction expectations in the social (*M* = 2.90, *SD* = 0.61) compared to the safety condition, (*M* = 2.60, *SD* = 0.67), *F*(1172) = 4.02, *p* = 0.047, η_p_^2^ = 0.023. No differences in expectancy estimates emerged between goal activation conditions among non-HED participants, both *p*s > 0.220.^1^

## Discussion

The current experiment extends the literature by examining how goal activation may not only increase, but also reduce, motivations and cognitions that predict college HED. Heavy drinkers primed with alcohol-congruent goals exhibited greater implicit alcohol approach and tension reduction expectancies compared to light drinkers. Conversely, heavy drinkers primed with alcohol-incongruent goals exhibited implicit alcohol avoidance, suggesting driving safety goals may activate negative alcohol-related associations. Supporting this assumption, heavy drinkers reported greater cognitive/behavioural impairment expectancies after driving safety primes. While these findings replicate past research suggesting social primes promote positive alcohol attitudes (Sheeran et al., 2005; Webb et al., 2012), our results extend the literature by demonstrating how priming negative alcohol-related contexts may reduce pro-alcohol biases, particularly among heavy drinkers. Importantly, goal priming was effective on explicit and implicit measures, which each predict unique variance in health behaviours and likely capture separate, but weakly related, constructs (Reich et al., 2010).

While our manipulation was adapted from previous research, we did not assess or pilot whether manipulations facilitated goal-state activation (e.g. immediate desire for socialising or safety). Thus, further research is needed to clearly delineate the precise mechanism underlying our findings. However, our results are in line with hypotheses and our methodology is consistent with research which has successfully facilitated goal-state activation (Ferguson, 2008; Sheeran et al., 2005; Webb et al., 2012). While we did not include follow-up assessment of drinking behaviour, implicit alcohol motivations are known to predict future alcohol use (Farris et al., 2010). While we expected social primes to impact a variety of positive expectancies, the lack of a priming effect on the social subscale of the CEOA was not anticipated. However, students in the present study – reflecting demographics on our urban, commuter campus – were older than participants in typical studies of college drinking, and therefore may hold different expectancies for social contexts. Further, the sample largely consisted of female participants. While college men and women may differ in patterns of alcohol use (Hoeppner et al., 2013), our findings suggest goal priming among heavy drinkers may be effective regardless of sex. However, future studies should examine how manipulations which target health cognitions, such as goal activation, differ across varying demographic subgroups.

Notwithstanding the limitations of the study, our findings are in line with studies reporting goal priming can alter both implicit and explicit attitudes (Ferguson and Porter, 2010; Fitzsimons and Shah, 2008). These findings integrate within the broader literature concerning how goal priming may influence health behaviours. While previous work has examined how social goal primes may promote pro-alcohol cognitions and behaviour (Sheeran et al., 2005; Webb et al., 2012), to our knowledge, this is the first published study to examine how priming driving safety goals may promote alcohol avoidance among heavy versus light drinkers. Further, while recent research has failed to replicate the effects of goal priming on cognitions and behaviour (Harris et al., 2013), our findings suggest individual differences (e.g. recent drinking history) may play a role in the effectiveness of goal priming. Consistent with Harris et al. (2013), we found no main effect differences between priming conditions. However, differences between prime conditions did emerge among heavy drinkers, suggesting goal priming may only be effective in changing alcohol-related cognitions among those reporting recent HED. Thus, our findings suggest the inclusion of individual and contextual factors that may moderate responses towards health-related behaviours following goal activation.

While these results have potential to contribute to the development of novel drinking interventions among heavy drinkers, additional work is needed to translate our findings into real-world contexts. For example, restaurants and grocery stores displaying health-related primes (e.g. advertisements, menus) seemingly promote healthier decision-making compared to when these primes are not present (Papies and Hamstra, 2010; Stöckli et al., 2016). Thus, future research may implement alcohol-incongruent primes in environments where HED may occur, examining their impact on alcohol use among heavy drinkers. Further, we did not capture the variety of alcohol-related goals in our manipulation. For example, future studies might examine the impact of priming alternative alcohol-related consequences (e.g. physical health, risk-taking). Additional research using our paradigm and other approaches to goal-priming (e.g. widening the range of goals), in addition to testing interactions between immediate goals and individual differences (e.g. recent drinking habits), may inform interventions to reduce college drinking.

## Supplemental Material

JHP_Supplementary_Material – Supplemental material for Don’t drink and drive, it’s a prime: Cognitive effects of priming alcohol-congruent and incongruent goals among heavy versus light drinkers Click here for additional data file. Supplemental material, JHP_Supplementary_Material for Don’t drink and drive, it’s a prime: Cognitive effects of priming alcohol-congruent and incongruent goals among heavy versus light drinkers by Zachary W Petzel and Jeffrey G Noel in Journal of Health Psychology

## Notes

1. Due to the overrepresentation of females, additional analyses were conducted with sex (male, female) as a factor. However, no factors significantly interacted with sex for implicit alcohol motivation, all *p*s > 0.195, or explicit alcohol expectancies, all *p*s > 0.233.
