# Supplementary material for: Don’t drink and drive, it’s a prime: Cognitive effects of priming alcohol-congruent and incongruent goals among heavy versus light drinkers
Source: J Health Psychol. 2020 Jun 12;26(14):2966–72. doi: 10.1177/1359105320934166 (PMC8543567; doi:10.1177/1359105320934166)
Supplement: JHP_Supplementary_Material – Supplemental material for Don’t drink and drive, it’s a prime: Cognitive effects of priming alcohol-congruent and incongruent goals among heavy versus light drinkers [file JHP_Supplementary_Material.pdf]

Safety Priming Questions

1. Do you currently drive or have you ever driven?
   a. Yes
   b. No

Please rate how often you do the following:

Rated from 1 (*Never*) to 4 (*Frequently*)

2. Wait to read incoming text messages until I reach my destination
3. Wait to eat food until I reach my destination
4. Try to limit distractions while driving
5. Drive with both hands on the wheel
6. Reduce my speed in work zones
7. Drive within 5 mph of the speed limit
8. Come to a complete stop at stop signs
9. Pass other cars on the left (rather than on the right side)
10. Change lanes to avoid cars pulled over on the shoulder
11. Use my turn signals
12. Come to a complete stop before turning right on red
13. Turn on my headlights when my windshield wipers are in use
14. Reduce my speed during hazardous driving conditions

The following items required open-ended responses:

15. How important is it to practice safe driving behaviors and habits? Explain.
16. Do you think it is common for adults to drink alcohol and then drive an automobile? Why or why not?

Social Priming Questions

Please rate how often you do the following:

Rated from 1 (*Never*) to 4 (*Frequently*)

1. See movies at the theater
2. Go shopping
3. Attend music concerts
4. Go out for happy hour
5. Attend professional sports games
6. Play on recreational sports teams
7. Talk on the phone

8. Videoconference using Skype, FaceTime, or another program
9. Text or instant message to chat
10. Play board games
11. Watch TV
12. Go for a walk / hike
13. Attend trivia nights
14. Volunteer for a charity

The following items required open-ended responses:

15. How important is it to have good friends? Explain.
16. Do you think it is common for adults to drink alcohol when they socialize with each other? Why or why not?
